# The effects of a Virtual Fracture Care review protocol on secondary healthcare utilization in trauma patients requiring semi-acute surgery: a retrospective cohort study

**DOI:** 10.3389/fdgth.2024.1362503

**Published:** 2024-06-17

**Authors:** G. J. A. Willinge, J. F. Spierings, T. H. Geerdink, B. A. Twigt, J. C. Goslings, R. N. van Veen

**Affiliations:** ^1^ Department of Trauma Surgery, OLVG Hospital, Amsterdam, Netherlands; ^2^ Department of Trauma Surgery, St. Antonius Hospital, Nieuwegein, Netherlands

**Keywords:** virtual fracture clinic, healthcare utilization, semi-acute surgery, telemedicine, extremity injuries

## Abstract

**Purpose:**

The demand for trauma care in the Netherlands is increasing due to a rising incidence of injuries. To provide adequate trauma care amidst this increasing pressure, a Virtual Fracture Care (VFC) review protocol was introduced for treatment of musculoskeletal injuries to the extremities (MIE). This study aimed to assess the influence of the Dutch VFC review protocol on secondary healthcare utilization (i.e., follow-up appointments and imaging) in adult trauma patients (aged ≥18 years) who underwent semi-acute surgery (2–14 days after initial presentation) for MIE, compared to traditional workflows. We hypothesized utilization of VFC review would lead to reduced secondary healthcare utilization.

**Methods:**

This retrospective cohort study assessed the influence of VFC review on secondary healthcare utilization in adult trauma patients (aged ≥18 years) who underwent semi-acute surgery for a MIE. Patients treated before VFC review and the COVID-19 pandemic, from 1st of July 2018 to 31st of December 2019, formed a pre-VFC group. Patients treated after VFC review implementation from January 1st 2021 to June 30th 2022, partially during and after the COVID-19 pandemic (including distancing measures), formed a VFC group. Outcomes were follow-up appointments, radiographic imaging, time to surgery, emergency department reattendances, and complications. The study was approved by the local ethical research committee approved this study (WO 23.073).

**Results:**

In total, 2,682 patients were included, consisting of 1,277 pre-VFC patients, and 1,405 VFC patients. Following VFC review, the total number of follow-up appointments reduced by 21% and a shift from face-to-face towards telephone consultations occurred with 19% of follow-up appointments performed by telephone in the VFC group vs. 4% in the pre-VFC group. Additionally, VFC review resulted in a 7% reduction of radiographs, improved time scheduling of surgery, and a 56% reduction of emergency department reattendances. Registered complication rates remained similar.

**Conclusion:**

The utilization of VFC review for management of adult patients with a MIE requiring semi-acute surgery improves efficiency compared to traditional workflows. It results in a 21% follow-up appointment reduction, a shift from face-to-face to remote delivery of care, fewer radiographs, improved time scheduling of surgery, and reduces emergency department reattendances by 56%.

## Introduction

In recent years, the rising incidence of musculoskeletal injuries of the extremities (MIE) has increased the pressure on the trauma care system in the Netherlands (NL) (1–3). From 2012 to 2022, the incidence of injuries scored as moderate to severe that is, an AIS score of 2+ (e.g., fractures), has risen by 16% and in 2022, approximately 36% (661.000) of all ED visits in NL were due to a traumatic injury (4). Two main driving forces for this trend are population growth and shifts in population demographics (5). Both younger and older age groups are expanding, coinciding with the peak incidence of injuries within these demographics. During the recent COVID pandemic, the strain on healthcare resources and medical personnel grew ever more urgent with less available personnel due to illness and infection risks, overcrowding in emergency departments (ED), and reallocation of resources and personnel to care for COVID-19 patients (6–8). Furthermore, treatment for MIE is gradually shifting from non-operative towards surgical management (3, 9). Due to the need for additional pre-operative and post-operative check-ups and imaging, this shift towards surgical treatment of MIE could potentially increase secondary healthcare utilization and thus further the increasing demand of trauma care. To address these challenges, reorganizing surgical treatment pathways and improving outpatient clinic management could alleviate the increasing pressure on Dutch trauma care.

One of the reorganizations in the management of patients with MIE is the Virtual Fracture Care (VFC) model, adopted by several countries in several variations (10–14). Generally, the VFC model consists of two parts: (1) a Direct Discharge protocol, through which patients with a simple and stable injury are directly discharged from the ED, and (2) a VFC review protocol, which utilizes an organized meeting to optimize follow-up treatment referral (14, 15). Additional to other VFC models, the Dutch version of the VFC review protocol extends beyond merely follow-up treatment referral. With this protocol, a multidisciplinary team digitally comprises a follow-up treatment plan (including all necessary appointments and imaging) focusing on uniform treatment, efficient planning of follow-up, and remote delivery of care, using pre-defined digital treatment plans. This plan is comprised for the complete follow-up treatment from start to finish. This approach is also used for patients requiring semi-acute surgery (scheduled surgery required in 2–14 days of initial presentation) and could potentially result in more efficient utilization of hospital resources and medical personnel. Previous studies on the efficiency of the VFC model have shown positive results regarding efficiency, cost-effectiveness and patient satisfaction but these studies mainly concerned the Direct Discharge protocol or VFC review protocols that only guide optimal patient referral (i.e., contrary to the Dutch version that includes complete digital follow-up treatment plans) (12–14, 16, 17).

Results on the efficiency of the Dutch VFC review protocol on the entire follow-up treatment pathways of patients with MIE requiring semi-acute surgery remain unknown. This knowledge could aid clinicians in optimizing these treatment pathways. Therefore, the primary aim of this study was to assess the influence of the Dutch VFC review protocol on secondary healthcare utilization (i.e., follow-up appointments and imaging) in adult trauma patients (aged ≥18 years) who underwent semi-acute surgery for MIE, compared to the traditional workflow. We hypothesized utilization of the VFC review protocol would lead to a reduction of secondary healthcare utilization for these patients.

## Materials and methods

### Design and setting

**Figure 1:**
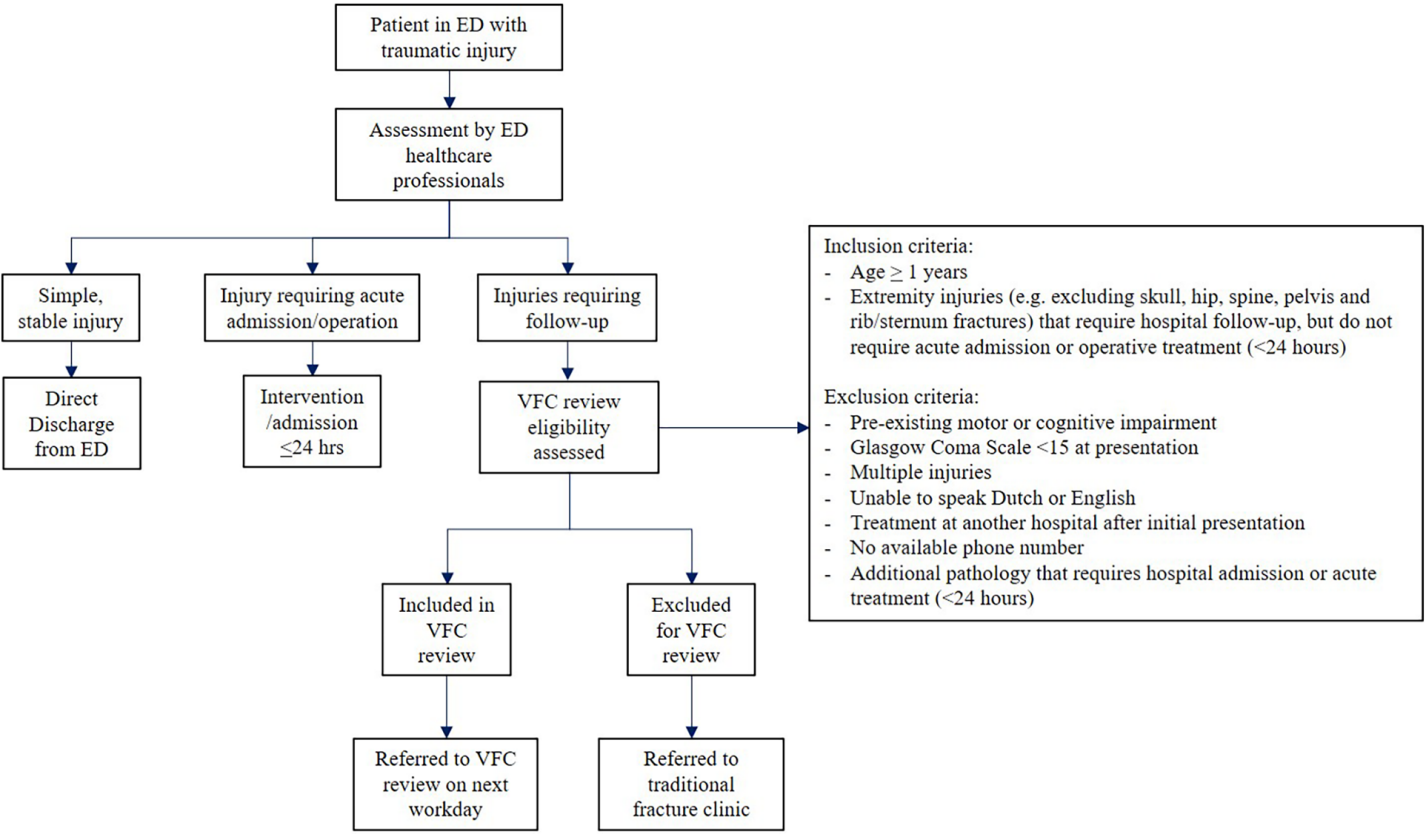
Flowchart of patient inclusion for VFC review. ED, emergency department; VFC, virtual fracture care.

This was a single-centre retrospective cohort study, performed at an urban level 2 trauma centre and teaching hospital in the Netherlands. A this hospital, a VFC review protocol was introduced in April 2020, and was implemented as standard care in January 2021 (15). With this new protocol, all Dutch or English speaking ED patients with an MIE aged at least one years old who required follow-up hospital treatment (e.g., both non-operative treatment or scheduled operative treatment) were treated according to the VFC review protocol (Figure 1). Exclusion criteria for VFC review were: indication for acute (≤24 h) surgical intervention or hospital admission (e.g., medical indication such as an open fracture, hemodynamic instability, severe soft-tissue damage, or a pending threat to soft-tissue, or social indication such as inability to be self-reliant at home or lack of support), pre-existing motor or cognitive impairment, Glasgow Coma Scale <15, multiple injuries, no available phone number, and initial treatment or follow-up elsewhere (due to the geographic location of the patients' residence or the patients' wish). The local ethical research committee approved this study (WO 23.073).

#### Study population

Patients were included in this study if they were aged ≥18 years, presented to the ED with a MIE that required semi-acute surgery (scheduled surgery required in 2–14 days of initial presentation) from 1st of July 2018—31st of December 2019 (pre-VFC group) and January 1st 2021—June 30th 2022 (VFC group), and were eligible for VFC review according to the stated inclusion and exclusion criteria. Indications for semi-acute surgery were based on national trauma treatment guidelines (e.g., displaced fracture, unstable fracture pattern, inability to successfully reduce the fracture, comminuted fracture). No other inclusion or exclusion criteria were used. The inclusion date for the VFC group was set on January 1st 2021 to account for the transition period after implementation of the new VFC review workflow. The inclusion period for VFC patients coincided with several COVID-19 restrictions. Supplementary Appendix S1 shows a timeline for the main COVID-19 related events and restrictions during this period (18). Notably, from the implementation of VFC review in 2021 to the removal of the final COVID-19 restriction in March 2022, the emphasis was placed on remote delivery of care whenever possible by the study institution board in response to governmental advice and policy during this period (18). However, no outpatient clinics were closed and face-to-face consultations were continuously allowed if deemed necessary by the treating physicians at the study institution. This included obligatory wearing of face masks and restrictions on accompanying visitors.

### Treatment workflows

#### Pre-VFC workflow

Before VFC review implementation, decision-making for surgical treatment was primarily done by (often inexperienced) residents in the ED, with varying levels of supervision by surgical staff. Treatment options were either discussed with the patient in the ED, or this was postponed to an appointment at the outpatient clinic or casting room shortly after the ED visit. Typically, these appointments were conducted by residents, with on-call supervision if deemed necessary. Additionally, to prevent incorrect diagnoses, musculoskeletal ED radiographs from the previous day were checked during a radiograph assessment meeting (attended by a radiologist, ED staff and a trauma surgeon) every workday. In case of an incorrect diagnosis, the patient was planned for an outpatient clinic appointment to discuss further treatment. After deciding on surgery, an administrative planner was electronically notified through the EPR and a date for the surgery was scheduled. The planner then notifies the patient of the definitive date of surgery. The scheduling process generally took 1–3 days. Further post-operative follow-up treatment was scheduled at each subsequent appointment by varying attending healthcare professionals. This process resulted in treatment variation and impaired uniform information provision and expectation management for patients. Furthermore, this potentially led to excessive follow-up appointments and imaging.

#### VFC—review workflow

**Figure 2:**
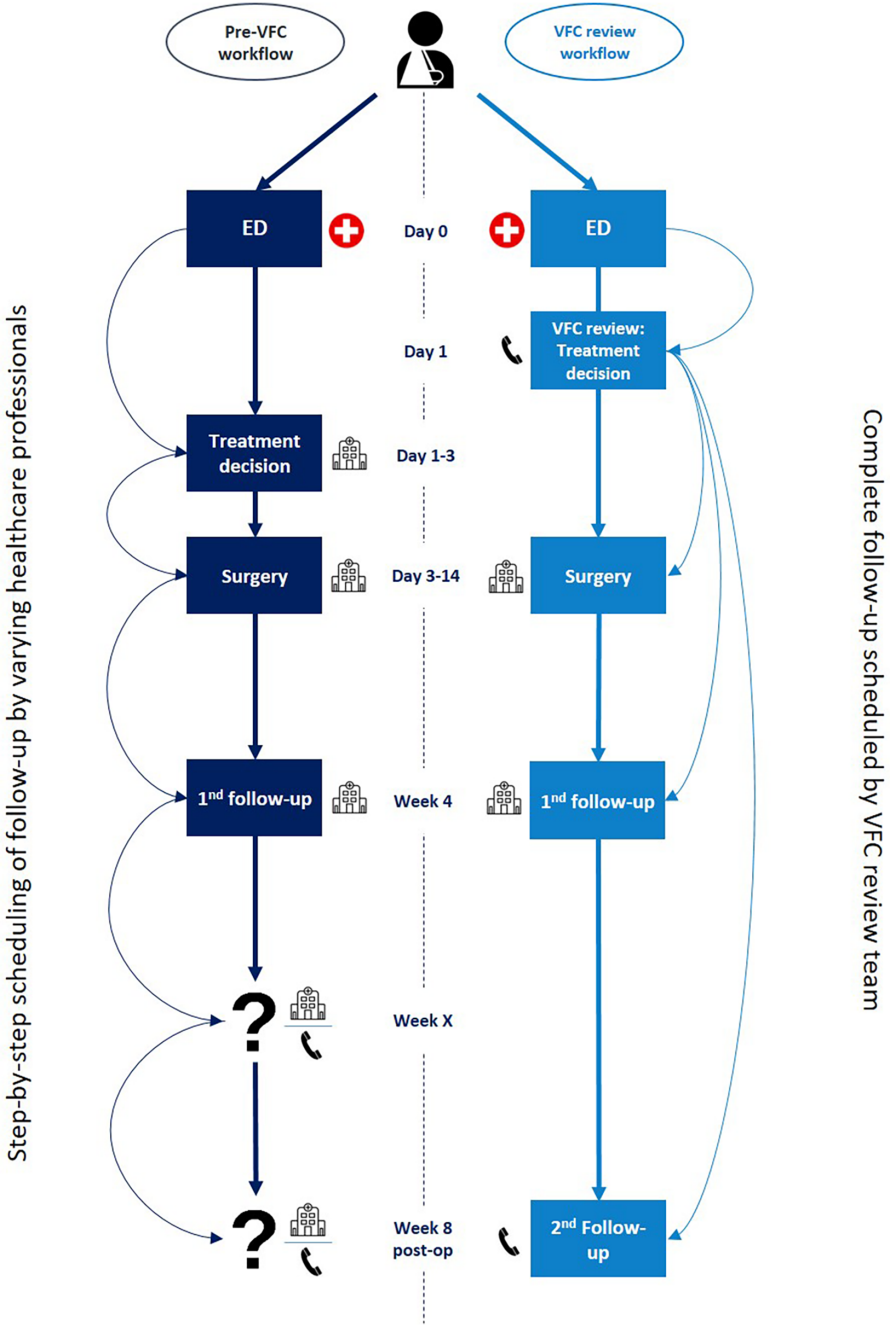
General overview of semi-acute surgical treatment pathways in both the pre-VFC and VFC review workflow. ED, emergency department; VFC, virtual fracture care.

The introduction of the VFC review brought about various modifications to the established workflow. Table 1 presents a summary of the differences between the pre-VFC and VFC workflows. Additionally, Figure 2 offers a chronological depiction of a typical semi-acute surgical treatment pathway in both the pre-VFC and VFC review workflow. With the VFC review workflow, ED healthcare professionals assessed VFC review eligibility and, if deemed eligible, referred patients to a VFC review meeting on the next workday. Patients were discharged from the ED and received digital and written information on the VFC workflow and their injury. To ensure all necessary patient information was available for VFC review, ED healthcare professionals were provided with orthopaedic pocket cards. These contained specific points of attention to document and instructions on when to perform an additional pre-operative CT scan.

**Table 1:** Pre-VFC vs. VFC workflow for patients requiring semi-acute surgical treatment.

Phases	Pre-VFC workflow	VFC—review workflow
Treatment protocols	• Local (hospital) and national orthopaedic trauma guidelines • Perspective at each follow-up appointment as far as the next follow-up appointment • Follow-up susceptible to variation between physicians • Information from multiple healthcare professionals, susceptible to misinformation, variation and confusion	• 180 complete multidisciplinary comprised treatment pathways, based on internal and external standards • Focus on remote delivery of care, reduction of unnecessary follow-up appointments and imaging, and uniform patient information provision • Uniform treatment and information provision from start to finish under direct supervision of experienced trauma surgeon • Treatment pathways digitally integrated into the EPR system and built into a separate interface. Using this interface, healthcare professionals could efficiently select and adjust each treatment plan for each patient as deemed appropriate through a series of dropdown menus.
ED visit	Timing: day 0	Timing: day 0
	• Initial care by ED healthcare professionals + consultation orthopaedic resident • Treatment discussed at the ED by resident, or postponed to fracture clinic appointment shortly (within 7 days) after ED visit (e.g., if the resident or required supervision is not readily available) • Discharge from ED after evaluation by orthopaedic resident • Additional pre-operative imaging at the outpatient clinic if necessary • Acute surgical intervention or hospital admission are always directly discussed at the ED with the orthopaedic consultant and treated accordingly	• Initial care by ED healthcare professionals • Check eligibility for VFC review by ED healthcare professionals according to in- and exclusion criteria • Patient electronically referred to the VFC review for the next workday • ED healthcare professionals receive instructions on the need for pre-operative imaging (e.g., CT-scan) and acquire this at the ED accordingly • Discharge from ED without consultation orthopaedic resident • Acute surgical intervention or hospital admission are always directly discussed at the ED with the orthopaedic consultant and treated accordingly
Surgical treatment planning	Timing: day 3–5	Timing: day 1
	• Final decision on treatment made at the ED or fracture clinic after ED visit • An administrative planner is electronically notified through the EPR and a date for the surgery is scheduled. The scheduling process generally takes 1–3 days. The planner then notifies the patient of the definitive date of surgery • Post-operative treatment planned by the varying healthcare professionals at each subsequent follow-up appointment • The attending physician decides on further imaging and immobilization • Step-by-step planning: repeated scheduling of follow-up until treatment is considered finished by the treating physician	• A multidisciplinary team, led by an orthopaedic trauma surgeon, discusses referrals and documents a complete treatment plan, including surgery specifics, follow-up appointments and imaging, required immobilization and specific instructions for each follow-up appointment • Patient receives information on the complete treatment plan directly after VFC review by phone and consensus on definitive treatment is reached • A date for surgery is scheduled by the attending administrative planner directly after consent is reached. The planner then notifies the patient of the definitive date of surgery. • Post-operative follow-up treatment performed according to the VFC review treatment plan from start to finish. Should any complications arise during follow-up, deviation from the scheduled treatment plan is discussed with the VFC review supervisor

VFC, virtual fracture care; ED, emergency department; CT, computed tomography; EPR, electronic patient record.

During the VFC review meeting, a multidisciplinary team [comprising a casting technician, surgical resident, (orthopaedic) trauma surgeon and administrative assistant] discussed all referrals and assigned treatment plans to each patient, using pre-defined digital treatment plans. A template for these treatment plans is shown in Supplementary Appendix S2. In total, 180 digital pre-defined treatment plans designed for each injury and required treatment (e.g., a distal radius fracture requiring operative treatment or a metatarsal shaft fracture requiring non-operative treatment) were established based on national fracture treatment guidelines and joint consensus of orthopaedic trauma staff, and were integrated into the EPR system based on this template. These complete treatment plans were readily accessible for the VFC team through a VFC interface within the EPR system and focused on matching patient needs at each follow-up appointment to the expertise of selected healthcare professionals, efficiently allocating and distributing tasks for each injury and treatment. Additionally, VFC review treatment plans focused on remote delivery of care and optimal uniform patient information provision (19). For surgical treatment, the VFC review treatment plans included information on the surgical procedure, the complete follow-up treatment and the recovery process (as illustrated in Supplementary Appendix S2). Prior to the VFC review, the attending resident prepares suggested treatment plans for each patient using the pre-defined treatment plans, which can then be efficiently checked and supervised by the complete VFC team during the VFC review meeting. The resident presents each case, the casting technician advices on the possibilities for immobilization and required follow-up in the casting room, and the supervising (orthopaedic) trauma surgeon makes the final decision on the most appropriate treatment plan. All required treatment components are then selected and documented through a series of dropdown menus, customizing the plan to fit each patient's needs. The VFC team (led by the orthopaedic trauma surgeon) could adjust the pre-defined treatment plans for each patient based on expert opinion, adding or deleting treatment components if deemed necessary. During VFC review, the attending surgical resident documents the treatment plans for each patient within the EPR, directly entering and saving data to each patient's record. This process resulted in an individualized, comprehensive, supervised and digital plan for surgery and follow-up treatment within one workday after the initial ED visit.

Directly after VFC review, all patients who required operative treatment were informed by phone by the VFC review supervisor to discuss the treatment options and reach consent on definitive treatment. If consent on definitive treatment could not be reached during the VFC phone call, patients were scheduled for an additional follow-up appointment by phone or at the outpatient clinic to discuss treatment options and reach consent on treatment at that time. For patients requiring surgery, an administrative planner scheduled the surgical procedure and all follow-up appointments after consent was reached, and notified the patient the same day via mail or their electronic patient record. Should any deviations from the VFC review treatment plan be required during follow-up treatment due to unforeseen circumstances (e.g., new trauma, wound infection, delayed fracture recovery, this is always consulted with the VFC review supervisor for that patient to make the necessary changes to the treatment plan.. If this supervisor is not available, the supervising orthopaedic trauma surgeon at that time is consulted.

### Data collection

The data for this study were anonymously extracted from an existing database. This database was set up as part of a clinical audit of the VFC review protocol performed in our institution and included patient records from patients treated between 2016 and 2023. Data in this database were directly extracted from EPR using a query designed to only include patients who were eligible for the VFC review workflow, both before and after VFC implementation to ensure comparability between groups. The query included registered patient characteristics in the EPR and detailed texts extracted from ED and outpatient clinic documentation.

For all study patients included from the pre-existing database, an additional manual check was performed by one of the researchers with extensive experience with the VFC review protocol (GW) to ensure VFC review eligibility before study inclusion and data extraction. If a patient was not eligible for VFC review, this patient was excluded from the study. The following data were extracted for the purposes of this study: age, sex, registered injury type (fracture, luxation, tendon/ligament rupture), registered diagnosis related group (DRG) (20), outpatient clinic follow-up appointments (face-to-face or remotely by phone) with each healthcare professional contact counting as one appointment (e.g., a combined appointment in the casting room with a physician and a casting technician, was counted as two separate appointments), type of healthcare professionals performing follow-up appointments (physician or casting technician), imaging during treatment (radiograph, computed tomography (CT) scan and magnetic resonance imaging (MRI) scan), time from first ED presentation to surgery (days), ED reattendances and registered complications. All data were collected for a period of up to one year after initial ED presentation for each patient.

To evaluate adequate timing of surgery, patients were divided into two groups based on the suggested optimal timeframe for surgery per injury type in the VFC review treatment plans. One group of injuries had a suggested optimal timeframe of ≤7 days post-injury, the second group had a suggested optimal timeframe of ≤14 days post-injury. These timeframes were based on joint consensus of orthopaedic trauma staff, guided by available literature on surgical timing and fracture guidelines (21–24). The grouping of specific injuries in these suggested optimal timeframes for surgery was reported at baseline.

### Outcomes

The primary outcome was the number and type of outpatient clinic follow-up appointments (face-to-face or remote), including the number and type of healthcare professionals performing these follow-up appointments.

Secondary outcomes included radiographic imaging (in the ED and outpatient clinic), the number of surgeries performed within their suggested optimal timeframe (days from ED presentation to surgery), ED reattendances and registered complications (e.g., wound infection, bleeding, wound dehiscence, nerve damage, failed osteosynthesis).

### Statistical analysis

The mean or median with appropriate measures of dispersion (standard deviation [SD] or interquartile range [IQR]) were used to present descriptive data for continuous variables. For categorical variables, frequencies and percentages were used. The Chi-squared test and the Mann-Whitney U test were applied to compare categorical and continuous data between groups at baseline. A *p-value* below 0.05 was deemed statistically significant. Additionally, regression analyses were used to account for potential differences in baseline characteristics and evaluate the independent effect of VFC review. For count data outcomes (e.g., follow-up appointments, imaging) Poisson regression and negative binomial regression were used depending on variable distribution (Poisson or overdispersed) (25). These outcomes were presented as incidence rate ratios (IRR) using the pre-VFC as a reference group. To illustrate: an IRR of 1.50 can be interpreted as an increased rate of 1.5 (50%) in the outcome parameter in the VFC group, compared to the pre-VFC group, whereas an IRR of 0.50 represents a decreased rate of 0.5 (−50%). Additionally, accessory confidence intervals and *p*-values were provided. For binary outcome data (surgery within suggested optimal timeframe yes/no, complication yes/no), a binary logistic regression analysis was performed. These outcomes were presented as odds ratios (OR) with pre-VFC patients as a reference group (26).

All regression analyses included the following factors and covariates: age, sex, registered injury type and registered DRG. Multiple imputation was not employed; missing data were excluded from analysis if encountered. Data was analysed using SPSS software, version 22 (IBM) (27).

## Results

In total, 2,682 patients were included: 1,277 pre-VFC patients, and 1,405 VFC patients (Table 2).

**Table 2:** Baseline characteristics of pre-VFC and VFC patients.

Variables	Pre-VFC (*n* = 1,277)		VFC (*n* = 1,405)		*p-value*
Sex: *n* (%)					*0*.*23*
Male	712	(56)	751	(54)	* *
Female	565	(44)	654	(46)	* *
Age in years: median (IQR)	40	(27–58)	41	(27–57)	*0*.*68*
Registered injury type: *n* (%)					*<0*.*01*
Fracture	1,207	(94)	1,282	(91)	* *
Luxation	24	(2)	51	(4)	* *
Tendon/ligament rupture	46	(4)	71	(5)	* *
Registered DRG: *n* (%)					
AC/SC joint	2	(0)	6	(1)	*0*.*20*
Clavicle	107	(8)	132	(9)	*0*.*36*
Glenohumeral/proximal humerus	39	(3)	52	(4)	*0*.*36*
Distal humerus/elbow	93	(7)	114	(8)	*0*.*42*
Radial head	32	(3)	15	(1)	*<0*.*01*
Radial/ulnar shaft	42	(3)	30	(2)	*0*.*06*
Distal radius/ulna	243	(19)	332	(24)	*<0*.*01*
Carpus	11	(1)	14	(1)	*0*.*72*
Metacarpus	123	(9)	120	(9)	*0*.*33*
Phalanges finger	148	(12)	142	(10)	*0*.*22*
Patella	29	(2)	22	(2)	*0*.*18*
Tibial plateau	36	(3)	33	(2)	*0*.*44*
Tibia/fibula shaft	36	(3)	33	(2)	*0*.*44*
Ankle	252	(20)	284	(20)	*0*.*76*
Achilles tendon	30	(2)	47	(3)	*0*.*12*
Tarsus	7	(1)	3	(0)	*0*.*21*
Metatarsus	32	(3)	20	(1)	*0*.*04*
Phalanges foot	15	(1)	6	(1)	*0*.*03*
Suggested optimal timeframe for surgery: *n* (%)					*0*.*94*
≤7 days^a^	817	(64)	901	(64)	* *
≤14 days^b^	460	(36)	504	(36)	

VFC, virtual fracture care; IQR, interquartile range; DRG, diagnosis related group; AC, acromioclavicular; SC, sternoclavicular.

^a^ Injuries with a suggested optimal timeframe for surgery of ≤7 days post-injury: AC/SC joint, humerus, radius/ulna, carpus, metacarpus, phalanges finger, Achilles tendon, tarsus, metatarsus, phalanges foot.

^b^ Injuries with a suggested optimal timeframe for surgery of ≤14 days post-injury: Clavicle, patellar, tibial, ankle injuries.

The groups differed at baseline in registered injury types and registered DRGs. For both groups, fractures were the most common injury type. The ankle and distal radius/ulna DRG were most frequently registered in both groups.

The total number of follow-up appointments was reduced by 21% following VFC review implementation (IRR = 0.79, 95% CI = 0.76–0.82, *p < 0.01*) (Table 3). Furthermore, the mode of care delivery shifted from face-to-face towards remote care, with a total reduction of face-to-face appointments of 33% (IRR = 0.67, 95% CI = 0.64–0.70, *p < 0.01*) in the VFC group compared to the pre-VFC group. In the VFC group, 19% of follow-up appointments was done remotely vs. 4% in the pre-VFC group (*p* < 0.01). Casting technicians performed a relatively higher percentage of follow-up appointments in the VFC group (69%) compared to the pre-VFC group (65%) (*p* < 0.01). Nevertheless, due to the 21% reduction of total follow-up appointments, VFC patients still had relatively fewer follow-up appointments with both casting technicians (IRR = 0.92, 95% CI = 0.86–0.98, *p < 0.01*) and physicians (IRR = 0.74, 95% CI = 0.71–0.77, *p < 0.01*) compared to pre-VFC patients.

**Table 3:** Secondary healthcare utilization for pre-VFC and VFC patients.

	Descriptive outcome				Effect (regression pre-VFC vs. VFC)		
Outcome; *n* (median, IQR)	Pre-VFC (*n* = 1,277)		VFC (*n* = 1,405)		IRR	95% CI	*p-value*
Total FU appointments	7,654	(5, 4–8)	6,532	(4, 3–6)	0.79*	0.76–0.82	*<0*.*01*
Face-to-face	7,344	(5, 4–7)	5,295	(3, 2–5)	0.67*	0.64–0.70	*<0*.*01*
By telephone	310	(0, 0–0)	1,237	(1, 0–1)	3.59*	3.15–4.09	*<0*.*01*
FU appointments per HP type							
Physician	5,275	(4, 3–5)	4,240	(3, 2–4)	0.74*	0.71–0.77	*<0*.*01*
Casting technician	2,379	(2, 1–3)	2,292	(1, 0–3)	0.92*	0.86–0.98	*<0*.*01*
Total radiographs	4,250	(3, 2–4)	4,328	(3, 2–4)	0.93**	0.89–0.98	*<0*.*01*
FU radiographs	2,418	(2, 1–3	2,192	(1, 1–2)	0.83**	0.79–0.88	*<0*.*01*
ED radiographs	1,832	(1, 1–2)	2,136	(1, 1–2)	1.07**	1.01–1.14	*0*.*05*
Total CT scans	649	(0, 0–1)	771	(1, 0–1)	1.08**	0.97–1.20	*0*.*14*
FU CT scans	274	(0, 0–0)	233	(0, 0–0)	0.77**	0.65–0.93	*<0*.*01*
ED CT scans	375	(0, 0–1)	538	(0, 0–1)	1.30**	1.14–1.49	*<0*.*01*
Total MRI scans	26	(0, 0–0)	20	(0, 0–0)	0.70**	0.37–1.21	*0*.*23*
FU MRI scans	26	(0, 0–0)	20	(0, 0–0)	0.70**	0.37–1.21	*0*.*23*
ED MRI scans	0	(0, 0–0)	0	(0, 0–0)	–	–	*–*
ED reattendances	436	(0, 0–1)	200	(0, 0–0)	0.43*	0.37–0.52	*<0*.*01*

VFC, virtual fracture care; IQR, interquartile range; IRR, incidence rate ratio; CI, confidence interval; FU, follow-up; HP, healthcare professional; ED, emergency department; CT, computed tomography; MRI, magnetic resonance imaging.

* Analysed with a negative binomial regression model with a reference value of 1 for the pre-VFC group.

** Analysed with a Poisson regression model with a reference value of 1 for the pre-VFC group.

In total, fewer radiographs were performed in the VFC group compared to the pre-VFC group, with a reduction of 7% (IRR = 0.93, 95% CI = 0.89–0.98, *p < 0.01*) (Table 3). This total reduction was the result of a 7% increase of radiographs performed in the ED (IRR = 1.07, 95% CI = 1.01–1.14, *p = 0.05*) vs. a 17% reduction of radiographs performed in the outpatient clinic (IRR = 0.83, 95% CI = 0.79–0.88, *p < 0.01*) following VFC review implementation. The VFC review protocol did not significantly influence the total number of performed CT scans (IRR = 1.08, 95% CI = 0.97–1.20, *p = 0.14*). However, the location of where CT scans were performed shifted from the outpatient clinic to the ED. The number of outpatient clinic CT scans decreased by 23% (IRR = 0.77, 95% CI = 0.65–0.93, *p < 0.01*) whereas the number of ED CT scans increased by 30% (IRR = 1.30,95% CI = 1.14–1.49, *p < 0.01*). The number of MRI scans did not differ between groups in both the outpatient clinic and the ED.

For time to surgery, subgroup analysis showed that patients with a suggested optimal timeframe to surgery of ≤7 days were treated earlier in the VFC group, with a median of 7 (IQR:5–9) days, compared to of 8 (IQR:6–11) in the pre-VFC group (*p* < 0.01). This led to a greater proportion of patients operated within the suggested 7-day timeframe in the VFC group, reaching 57%, in comparison to 44% in the pre-VFC group (Table 4). Regression analysis showed that VFC patients had 1.79 (OR = 1.79, 95% CI = 1.45–2.20, *p < 0.01*) times higher odds of undergoing surgery within the suggested 7-day timeframe compared to pre-VFC patients. Contrarily, patients with a suggested optimal timeframe of ≤14 days to surgery were treated later in the VFC group, with a median of 11 (IQR:9–13) days, compared to 10 (IQR:9–12) days in the pre-VFC group (*p* < 0.01). However, the proportion of patients who underwent surgery within the suggested 14-day timeframe remained similar at 91%. Consequently, the chance of undergoing surgery within the suggested 14-day timeframe was comparable between both groups (OR = 1.0, 95% CI = 0.64–1.56, *p = 0.98*).

**Table 4:** Surgery performed within suggested optimal timeframes for pre-VFC patients and VFC patients.

Outcomes	Descriptive outcome		Effect (Regression pre-VFC vs. VFC)				
Injuries with suggested optimal timeframe ≤7 days	Pre-VFC (*n* = 817)		VFC (*n* = 901)		OR*	95% CI	p-value
Surgery within timeframe; *n* (%)	362	(44)	509	(57)	1.79	1.45–2.20	<0.01
Injuries with suggested optimal timeframe ≤14 days	Pre-VFC (*n* = 460)		VFC (*n* = 504)		OR*	95% CI	p-value
Surgery within timeframe; *n* (%)	420	(91)	459	(91)	1.0	0.64–1.56	0.98

VFC, virtual fracture care; OR, Odds ratio; CI, confidence interval.

* Analysed with a binary logistic regression model with a reference value of 1 for the pre-VFC group.

ED reattendances more than halved in the VFC group compared to the pre-VFC group, with a reduction of 56% following VFC review implementation (IRR = 0.46, 95% CI = 0.37–0.52, *p < 0.01*) (Table 3). The number of registered complications was low and comparable in both groups, with 53 (3.8%) in the VFC group, compared to 39 (3.1%) in the pre-VFC group (OR = 1.31, 95% CI = 0.85–2.01, *p = 0.22*). Registered complications included wound infections, failed osteosynthesis, bleeding, wound dehiscence and nerve damage. No significant differences were observed for all types of complications.

## Discussion

Implementation of a VFC review protocol significantly reduced follow-up appointments in semi-acute surgical pathways for patients with MIE, compared to traditional treatment protocols. In addition, implementation of VFC review shifted the mode of care delivery from face-to-face towards remote care, reduced radiographic imaging and more than halved ED reattendances.

The reduction of follow-up appointments was primarily achieved by efficiently organizing follow-up treatment planning and limiting excessive treatment, similar to Direct Discharge protocols (14, 28). Before implementation of VFC, follow-up treatment was inconsistently scheduled step-by-step by varying (often inexperienced) residents, leading to limited perspectives, inefficient planning and treatment variation (29). The VFC protocol replaces this with a supervised process and a multidisciplinary review meeting for better treatment coordination. Furthermore, by matching the needs at each follow-up appointment to the required expertise, excessive utilization of healthcare professionals can be limited and a more efficient allocation of tasks is achieved. This allocation also explains the higher percentage of follow-up appointments performed by casting technicians. Many follow-up appointments in these treatment plans involve adjustments or removal of immobilisation material, suitable for a well instructed casting technician without routine presence of a physician. Furthermore, the documented VFC review treatment plans provide specific instructions to support healthcare professionals during follow-up. These results complement previous studies, suggesting this VFC review model enhances complete follow-up treatment pathways, beyond merely referring patients to the right healthcare professional in the initial treatment phase (16).

In line with current literature, our results suggest the VFC review protocol results in a shift from face-to-face to remote care (28, 30, 31). New technologies, catalysed by the recent COVID-19 pandemic, have sparked widespread utilization of remote delivery of care through telemedicine due to its efficiency and satisfactory results (32). Furthermore, previous studies have reported societal and environmental benefits following introduction of similar VFC protocols and remote care (33). This is mainly attributed to reduced patient movements from-and-to the hospital due to the reduction of face-to-face care. Our findings show this also applies to the use of a VFC review protocol for patients requiring semi-acute surgery and subsequent hospital follow-up treatment. Notably, this may only hold true in countries with well-established communication infrastructure and high cell-phone saturation rates.

The reduction in radiographs in the VFC group aligns with results from previous studies and can be attributed to the more organized and supervised decision-making process during the VFC review meeting, similar to the reduction of follow-up appointments (28, 34). Additionally, the introduction of this protocol has led to a shift in time and place for CT scans, from the follow-up treatment phase in the outpatient clinic towards the initial treatment phase in the ED. This shift may be explained by the additional instructions for ED healthcare professionals on when to perform a CT scan. As a result of this shift, CT scans were readily available during the subsequent review meeting allowing (earlier) interpretation by the VFC team. Besides earlier interpretation, this also has a logistic patient friendly advantage, as these patients (often with limited mobility) do not have to come back to the hospital for a CT-scan. However, this advantage only applies to hospitals with sufficient resources to perform CT scans in the ED.

In our study, patients with an injury benefitting from early surgery (suggested optimal timeframe of ≤7 days) were scheduled earlier after VFC review implementation, whereas patients who were able to wait longer (suggested optimal timeframe ≤14 days) were scheduled later. However, the latter group of patients still adhered to the set suggested optimal timeframe, highlighting the role of VFC review in prioritizing patients and optimizing surgical scheduling planning accordingly. To our knowledge, no other studies on VFC review protocols have addressed this. Nevertheless, it should be noted that the timely scheduling of surgery depends on more than just the planning process. Factors like operating room capacity and the availability of personnel can also play a role. In this regard, timely scheduling of surgery may have been impaired in part of the VFC group due to the COVID pandemic, during which operating rooms and medical personnel were less available. This potentially led to an underestimation of the beneficial influence of VFC reviews on the optimization of surgical planning. Notably, the effects of the adherence to these timeframes on post-operative outcomes is unclear for most injuries. Future studies are needed to specify the optimal time to surgery for specific injuries, which can then be used to optimize the VFC review treatment plans.

Both our results and previous literature show that patients treated through VFC were less likely to reattend the ED compared to pre-VFC treatment (13, 35). However, previous studies mainly focused on the Direct Discharge protocols, only including non-operative management of simple and stable injuries. Our study indicates this also applies to trauma patients requiring semi-acute surgery. We reckon this is primarily due to the extensive information provision following VFC review, as one of the main reasons for ED reattendance is uncertainty about the recovery progress and timing of surgery and follow-up appointments (36). Especially for patients requiring surgery, lack of information may cause anxiety and concern (37). Furthermore, the one-day time interval between the ED visit and the VFC review phone consultation allows patients more time to think about their concerns and questions, which they could then address during the VFC review consultation. This may have prevented patients from contacting the ED after their visit due to these concerns. Fewer ED reattendances contribute to a reduction of ED crowding, which is a major global healthcare issue and known to significantly affect quality of care (38). Our current study lacked data to evaluate the specific reasons for ED reattendances, as this was not registered in the clinical audit database from which the study data was extracted. Therefore, we could only report on the number of ED reattendances. Detailed evaluation of reasons for ED reattendances and patient experiences could complement these results and provide more in-depth insight into the specific reason behind this reduction.

Strengths of this study include the level of detail of secondary healthcare utilization analysis, accounting for every contact with diverse healthcare professionals during follow-up treatment. Furthermore, the analysis was corrected for potential confounding due to baseline differences. Combined, this provided a detailed perspective on the independent effect of VFC review on the study outcome parameters. This study also had several limitations. First, we did not assess the number of missed follow-up appointments, as these were not registered in the study database. By changing the scheduling process and mode of delivery for follow-up appointments, the number of no-shows may have been affected. However, previous studies have shown that telemedicine reduces no-show rates, specifically in surgically treated patients (39–41). Additionally, we could not account visits to healthcare professionals outside of the hospital. This may have resulted in an underestimation of primary healthcare resource utilization. Nonetheless, as all patients and their general physicians were emphatically instructed to contact the hospital in case of any concerns or questions both before and after VFC review implementation, it is unlikely this would have been affected. Another important limitation is the potential bias from the COVID pandemic, occurring partially during the VFC group inclusion period, as the pandemic also influenced a shift to remote care. However, the primary aim of VFC review was to streamline trauma care, ensuring equal patient management with consistent resources, beyond merely facilitating remote care during the pandemic. As our study's observed results are greater than just a shift to remote care, we believe these stem from aspects unaffected by the pandemic, such as organized supervision, multidisciplinary planning, and treatment uniformity. Additionally, a prior study comparing a similar VFC review workflow to pre-VFC treatment during the same COVID period in two hospital sites yielded positive results favoring the VFC review, reinforcing the attribution of outcomes to the VFC review workflow (42). Finally, the reduction of follow-up appointments, improved allocation of tasks and shift to remote care following VFC review suggests a concomitant cost reduction. However, our data was insufficient to analyse the balance between the resources the VFC review protocol requires and the benefits it yields in terms of cost-effectiveness.

Parallel to healthcare resource utilization, the VFC review may also affect patients perspectives, warranting further research in patient satisfaction and experiences. In this regard, the experiences of healthcare professionals with newly introduced workflows should also be considered, as these changes often significantly impact their daily routines. These insights can aid successful integration of new workflows into daily clinical practice. Furthermore, studies focusing on specific treatment pathways and patient journeys are needed. This knowledge may provide insights in how specific pathways can be further tailored to fit varying needs of patients with different injuries. For example, patients who have more anxiety regarding their recovery may prefer face-to-face consultation over teleconsultation. Incorporating such patient preferences could enhance patient-friendliness and fit of treatment. Finally, a detailed cost-effectiveness analysis would be needed to determine the effect of the VFC review protocol on societal and hospital costs.

## Conclusion

The utilization of a VFC review protocol for management of adult patients (aged ≥18 years) with MIE requiring semi-acute surgery improves efficiency compared to traditional treatment protocols. By utilizing unified treatment pathways, organized and detailed collective review of patients by a multidisciplinary team, and complete treatment scheduling, secondary healthcare utilization was optimized. This included a 21% reduction of follow-up appointments, a shift from face-to-face remote delivery of care, fewer radiographs, improved time scheduling of surgery, and a 56% reduction of ED reattendances. This knowledge may aid clinicians in optimizing follow-up treatment pathways, providing uniform treatment and maintain quality of care while alleviating the increasing pressure on Dutch trauma care.

## Data Availability

The data analyzed in this study is subject to the following licenses/restrictions: Study data were anonymously extracted from an exisiting dataset, which was comprised as part of an institutional clinical audit. This dataset is only accessible to those within our institution authorized for access. However, our anonymous study data can be made available upon reasonable request. Requests to access these datasets should be directed to g.j.a.willinge@olvg.nl.
