# Supplementary figures and images for: The effects of a Virtual Fracture Care review protocol on secondary healthcare utilization in trauma patients requiring semi-acute surgery: a retrospective cohort study
Source: Front Digit Health. 2024 Jun 17;6:1362503. doi: 10.3389/fdgth.2024.1362503 (PMC11215198; doi:10.3389/fdgth.2024.1362503)

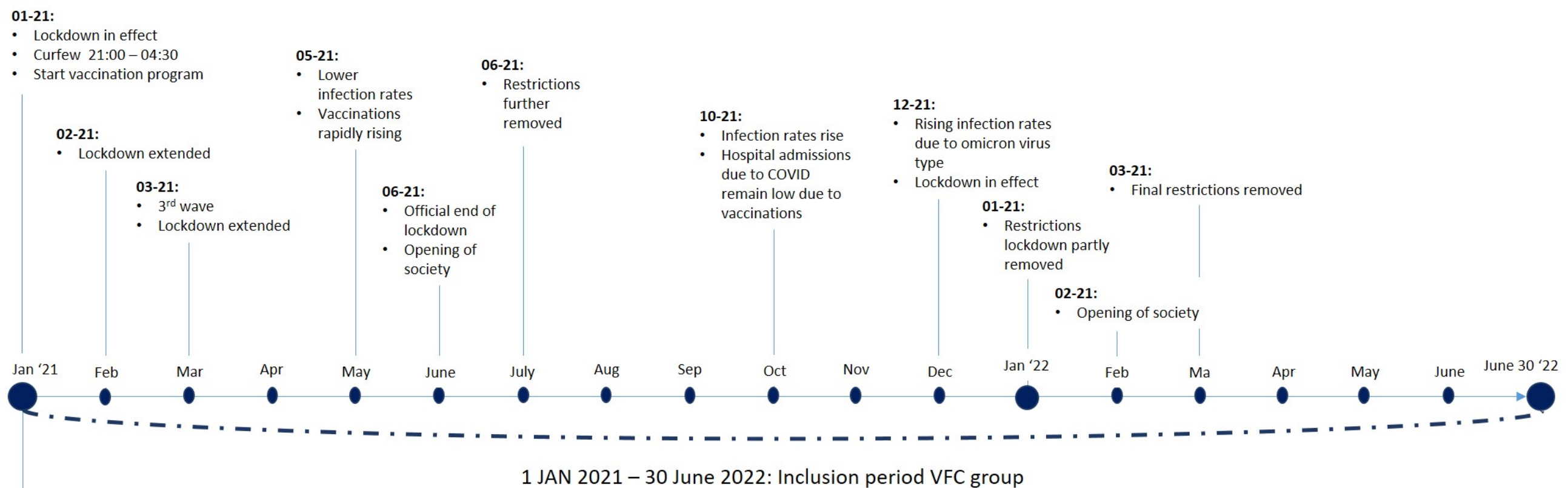

**01-21**

- VFC review implemented as new standard of care after 8-month pilot period

Supplement: Supplementary file 1 [file Datasheet1.pdf]
