# Supplementary material for: The effects of a Virtual Fracture Care review protocol on secondary healthcare utilization in trauma patients requiring semi-acute surgery: a retrospective cohort study
Source: Front Digit Health. 2024 Jun 17;6:1362503. doi: 10.3389/fdgth.2024.1362503 (PMC11215198; doi:10.3389/fdgth.2024.1362503)
Supplement: Supplementary file 2 [file Datasheet2.pdf]

Appendix 1

VFC review meeting
Supervisor: {select supervisor}
Diagnosis: {select diagnosis} {select right or left side}
Current immobilization: *ED immobilization method applied*

Suggested treatment plan
Operation plan: {free text to describe type of operation, approach and materials},
Estimated duration: *** minutes
Optimal planning: within *** days,
Surgical staff: {dropdown menu to choose desired surgical staff}
Pre-operative CT scan: {select necessity for pre-operative CT-scan}

Aim: *free text describing the main aim of the treatment*
Post-operative immobilization: *free text describing post-operative immobilization method*

Appointment	When	Location	Healthcare professional	Instructions	Radiographic imaging	Additional remarks
1	{select desired time}	{select location and mode of care delivery}	{select healthcare professionals}	*free text to provide specific instructions for healthcare professionals per appointment*	{type of radiographic imaging required}	*free text for additional remarks (e.g. need for physiotherapist referral)*
2	{select desired time}	{select location and mode of care delivery}	{select healthcare professionals}	*free text to provide specific instructions for healthcare professionals per appointment*	{type of radiographic imaging required}	*free text for additional remarks*
3	{select desired time}	{select location and mode of care delivery}	{select healthcare professionals}	*free text to provide specific instructions for healthcare professionals per appointment	{type of radiographic imaging required}	*free text for additional remarks
4	{select desired time}	{select location and mode of care delivery}	{select healthcare professionals}	*free text to provide specific instructions for healthcare professionals per appointment*	{type of radiographic imaging required}	*free text for additional remarks

Relevant information for the patient:
- *Free text describing the injury's general prognosis and potential complications*
- *Free text describing necessary immobilization method and duration*
- *Free text describing if and when to start exercises and if applicable physiotherapy*
- *Free text describing if and when to start physiotherapy*
- *Free text for additional points of attention during recovery*

The suggested treatment plan will be discussed with the patient by: {select healthcare professional}

Mandatory information for patients undergoing surgery as stated by the Act on Medical Treatment agreement:

- Type of surgery
- Complications
    - Early
    - Late
- Prognosis
- Alternative treatment method
